# The ProteomeXchange consortium in 2017: supporting the cultural change in proteomics public data deposition

**DOI:** 10.1093/nar/gkw936

**Published:** 2016-10-18

**Authors:** Eric W. Deutsch, Attila Csordas, Zhi Sun, Andrew Jarnuczak, Yasset Perez-Riverol, Tobias Ternent, David S. Campbell, Manuel Bernal-Llinares, Shujiro Okuda, Shin Kawano, Robert L. Moritz, Jeremy J. Carver, Mingxun Wang, Yasushi Ishihama, Nuno Bandeira, Henning Hermjakob, Juan Antonio Vizcaíno

**Affiliations:** 1 Institute for Systems Biology, Seattle, WA 98109, USA; 2 European Molecular Biology Laboratory, European Bioinformatics Institute (EMBL-EBI), Wellcome Trust Genome Campus, Hinxton, Cambridge, CB10 1SD, UK; 3 Niigata University Graduate School of Medical and Dental Sciences, Niigata 951-8510, Japan; 4 Database Center for Life Science, Joint Support-Center for Data Science Research, Research Organization of Information and Systems, Kashiwa 277-0871, Japan; 5 Center for Computational Mass Spectrometry, University of California, San Diego (UCSD), La Jolla, CA 92093, USA; 6 Department Computer Science and Engineering, University of California, San Diego (UCSD), La Jolla, CA 92093, USA; 7 Skaggs School of Pharmacy and Pharmaceutical Sciences, University of California, San Diego (UCSD), La Jolla, CA 92093, USA; 8 Graduate School of Pharmaceutical Sciences, Kyoto University, Kyoto 606-8501, Japan; 9 National Center for Protein Sciences, Beijing, China

## Abstract

The ProteomeXchange (PX) Consortium of proteomics resources (http://www.proteomexchange.org) was formally started in 2011 to standardize data submission and dissemination of mass spectrometry proteomics data worldwide. We give an overview of the current consortium activities and describe the advances of the past few years. Augmenting the PX founding members (PRIDE and PeptideAtlas, including the PASSEL resource), two new members have joined the consortium: MassIVE and jPOST. ProteomeCentral remains as the common data access portal, providing the ability to search for data sets in all participating PX resources, now with enhanced data visualization components.

We describe the updated submission guidelines, now expanded to include four members instead of two. As demonstrated by data submission statistics, PX is supporting a change in culture of the proteomics field: public data sharing is now an accepted standard, supported by requirements for journal submissions resulting in public data release becoming the norm. More than 4500 data sets have been submitted to the various PX resources since 2012. Human is the most represented species with approximately half of the data sets, followed by some of the main model organisms and a growing list of more than 900 diverse species. Data reprocessing activities are becoming more prominent, with both MassIVE and PeptideAtlas releasing the results of reprocessed data sets. Finally, we outline the upcoming advances for ProteomeXchange.

## INTRODUCTION

Life sciences as a whole has a strong tradition of open data dissemination. Prime examples of long-running and successful consortia to systematically collect, exchange and disseminate biomolecular data are the International Nucleotide Sequence Database Collaboration (1) for DNA sequence data, and the worldwide Protein Data Bank (2) for protein structure data. However, in some other life science domains, open data sharing has been for a long time more the exception than the norm. Yet in recent years, major initiatives in these other domains have successfully expanded the practice of public data sharing.

One such example is the ProteomeXchange (PX) Consortium (3) (http://www.proteomexchange.org), devoted to mass spectrometry (MS)-based proteomics data. Proteomics data resources such as PRIDE (4) (http://www.ebi.ac.uk/pride, EMBL-EBI, European Bioinformatics Institute, Cambridge, UK) and PeptideAtlas (5) (http://www.peptideatlas.org, Institute for Systems Biology, Seattle, WA, USA) had accepted submissions of proteomics data sets and handled MS proteomics data for many years. Yet, until the formation of the PX Consortium, they had been acting independently with very limited global coordination.

The implementation of the PX Consortium formally started in 2011 with the overall objective to provide a common framework and infrastructure for the cooperation of proteomics resources by defining and implementing consistent, harmonized, user-friendly data deposition and exchange procedures among the members. In the first stable implementation of the data workflow (3), PRIDE (now called PRIDE Archive) was the point of submission for tandem MS experiments (the most popular experimental approach in the proteomics field), while PeptideAtlas provided a repository for SRM (Selected Reaction Monitoring) experiments called PASSEL (PeptideAtlas SRM Experiment Library) (6). A common data access portal called ProteomeCentral was also developed (http://proteomecentral.proteomexchange.org), providing the ability to search for data sets in all participating PX resources. This functionality was made possible since the PX partners agreed to provide a baseline set of experimental and technical metadata for all data sets, which is encoded in the PX XML format (3).

There are other resources outside PX dedicated to the reanalysis of MS proteomics data, for instance GPMDB (7) and proteomics DB (8). For a complete overview of the existing proteomics resources, see (9). Based on rapidly increasing availability of open data, reuse of public proteomics data is flourishing in the field (see (10)), as exemplified in one of the drafts of the human proteome published in *Nature* (8). To facilitate data reuse and reanalysis, the PX partners fully support the open data formats developed under the umbrella of the Proteomics Standards Initiative (PSI) (11) and actively develop and maintain open-source software to support the standards (12).

Here, we will provide an update of the ProteomeXchange consortium since the original paper was published in 2014 (3), including a description of the updated submission guidelines, which now reflect PX's expansion to four consortium members. We will also describe the improvements in the common interface at ProteomeCentral, highlight different statistics to demonstrate the wide adoption of PX in the field and discuss future developments.

## EXPANSION OF THE CONSORTIUM AND UPDATED SUBMISSION GUIDELINES FOR ORIGINAL DATA SETS

Since the original implementation of the PX data workflow, two new resources have joined PX: the MassIVE repository (University of California San Diego, CA, USA, http://massive.ucsd.edu) joined in June 2014, and the recently developed jPOST resource (several institutions, Japan, http://jpost.org/) has just joined PX in July 2016, thus demonstrating PX's unifying role in the proteomics community by inclusion of members that were not part of the initial consortium. The jPOST system was developed based on the semantic web technology using the resource description framework data model.

The PX receiving repositories mentioned above (PRIDE, PASSEL, MassIVE and jPOST) store MS proteomics data as initially produced and analyzed by the scientists and provide private access for reviewers and journal editors during the manuscript review process. All the submitted data sets are assigned a unique and universal identifier of the format PXDnnnnnn (http://www.ebi.ac.uk/miriam/main/collections/MIR:00000513), although repository-specific identifiers can also be provided. The updated submission guidelines are available at http://www.proteomexchange.org/docs/guidelines_px.pdf.

There are two main data submission workflows, called ‘Complete’ and ‘Partial’. For both types, a set of common experimental metadata (encoded in the PX XML format), raw data mass spectra and the submitter's results are always mandatory. The difference lies in the way the processed peptide and protein identification results are handled. In the case of a ‘Complete’ data set, it is possible for the receiving PX repository to parse, ingest and directly connect the identification results with the submitted mass spectra. This can be achieved if the processed results and the corresponding spectra are available in supported open data formats. In some cases, it is also possible to use repository interfaces to assist with the conversion of files from proprietary formats to the supported open formats (e.g. MassIVE supports online conversion of Thermo RAW files to standard mzML (13) files).

On the other hand, ‘Partial’ submissions contain result files that are not in formats that can be parsed, and thus ingested, by the receiving repository. The metadata still make the data sets findable in the receiving repository and in ProteomeCentral. These data sets may then be freely downloaded and interpreted if the downloader has suitable software to parse the files. Or the data may be reprocessed from the mandatory raw data. This mechanism is needed for the output of tools not supporting open standards (see above), which is mainly the case for data workflows different from data dependent acquisition (DDA) approaches. Instead, the corresponding results files output by the non-compliant software tools (in heterogeneous formats) are made available for download. Thus, using the ‘Partial’ submission mechanism, virtually any type of proteomics data workflows can be supported. In fact, PX resources store a significant number of data sets coming from other data workflows such as Data Independent Acquisition (DIA) approaches, top-down proteomics or MS imaging (14), with MassIVE already supporting ‘complete’ submissions for some DIA workflows, such as MSPLIT-DIA's (15) analysis of SWATH-MS files.

**Figure 1.**
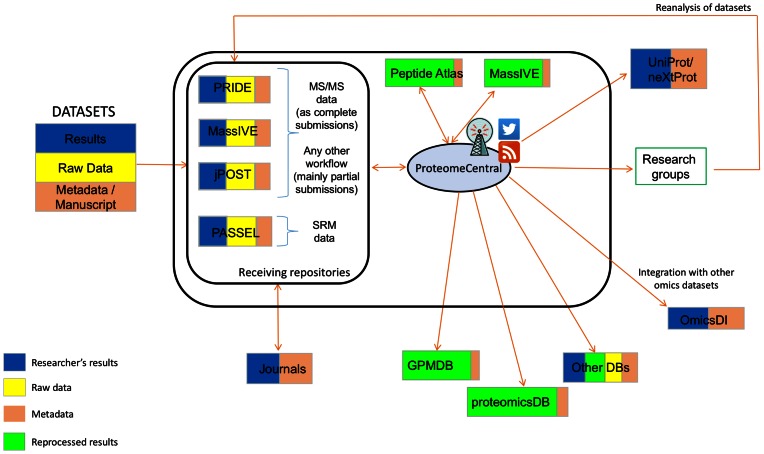
Schematic representation of the ProteomeXchange data workflow.

The overall organization of the Consortium is shown in Figure 1. Within PX, PRIDE, MassIVE and jPOST are considered to be *Universal* Archival resources, and at present they are focused on supporting tandem MS DDA workflows as ‘complete’ submissions. The PSI data standard for peptide and protein identification data, called mzIdentML (16) (plus the corresponding mass spectra), is the preferred format to perform ‘complete’ submissions. In fact, data submissions in this format are becoming easier since mzidentML is increasingly supported by the most popular proteomics analysis and visualization software (see details at http://www.psidev.info/tools-implementing-mzidentml), although there are still some notable exceptions, mainly MaxQuant and ProteomeDiscoverer^®^ (Thermo Fisher Scientific, Waltham, MA, USA), at the moment of writing. PRIDE still supports PRIDE XML as a submission format for ‘complete’ submissions as well, although submissions in this format are only encouraged if there is no feasible alternative for generating mzIdentML files. Once support for mzIdentML is generalized, PRIDE will discontinue support for PRIDE XML due to the limitations of the format. Another PSI data standard, called mzTab (17), stores identification and quantification data in a tabular format, and is supported by MassIVE and jPOST for performing ‘complete’ submissions. In MassIVE, online conversion tools are available to support conversion from tab-separated-value (TSV) formats into mzTab. PRIDE plans to formally support mzTab in the first half of 2017. In PRIDE, all ‘complete’ data sets also get a digital object identifier, to improve the tracking of data sets and improve recognition for submitters. PASSEL, the remaining PX Archival resource, is a *focused* resource. As mentioned in the introduction, it supports the submission of data coming from SRM workflows, but not other data workflows. In this case, the other PX members do not actively solicit this type of data, and recommend submission to PASSEL. It is important to note that, although recommended, this policy cannot be completely enforced since the submitter chooses which resource to use for deposition. See Table 1 for an overview of the workflows that each PX resource supports.

**Table 1.** Summary of submission guidelines for each PX resource, depending on the data workflow involved

	PRIDE	PASSEL	MassIVE	jPOST
**DDA MS/MS**				
Partial	Yes	No	Yes	Yes
Complete: mzIdentML	Yes	No	Yes	Yes
Complete: mzTab	No	No	Yes	Yes
Complete: TSV	No	No	Yes	No
Complete: PRIDE XML	Yes	No	No	No
**Other workflows**				
Targeted SRM/MRM	Partial only	Partial and complete	Partial only	Partial only
DIA MS/MS	Partial only	No	Partial and complete	Partial only
Top-down	Partial only	No	Partial only	Partial only
Mass spectrometry imaging	Partial only	No	Partial only	Partial only

Every PX resource has an independent data submission platform. PRIDE uses a Java-based open source stand-alone tool (the ‘PX submission tool’), whereas the other resources have web-based platforms for submission of data sets. In the web interfaces of the different PX resources, users can interactively search for peptides and protein identifications, considering different metadata filters and criteria. Mass spectra are also made accessible and visualized via the web interface, apart from the case of PRIDE, which plans to make this possible in the coming months.

The PRIDE team also actively develops a stand-alone visualization and data analysis tool called PRIDE Inspector (18,19), that supports the PSI data standards mzIdentML, mzTab and mzML (13), PRIDE XML and other open data formats. The tool can be used to visualize and check the data before submission (by the submitter), during the review process by reviewers and editors and after the data is made publicly available (by everyone).

## REPRESENTATION OF REPROCESSED DATA SETS

In addition to original data submissions, PX supports the storage and tracking of reprocessed (re-analyzed) versions of data sets that were originally submitted to one of the PX resources. These data sets get an identifier of the format RPXDnnnnnn and are also made available through ProteomeCentral. This mechanism is currently illustrated by a series of PeptideAtlas RPXD submissions reanalyzing several PX data sets, which are used in species- or organ-specific builds of PeptideAtlas resources (see http://www.peptideatlas.org/builds/). In addition to this, MassIVE has recently started a large-scale effort to provide complete reanalysis using a baseline standard data analysis workflow and will soon substantially increase the volume of reanalysis results. For example, the first reanalysis of one of the human draft proteomes published in *Nature* (20) is already available at MassIVE for the corresponding data set PXD000561 (see ‘Data set Reanalyses’ section on the MassIVE data set page at http://massive.ucsd.edu/ProteoSAFe/result.jsp?task=fd246a746e0749c5ad0403be265bb2ea&view=advanced_view). While we readily agree that no baseline reanalysis should be taken as a ‘standard of truth’, it is important that a well-characterized search approach be applied to as much data as possible to allow: (i) for comparison to submitted results; and (ii) for comparison of results between related data sets. In all RPXD data sets, the original source of the data is always properly cited and acknowledged.

In addition to reprocessing data sets, MassIVE also provides online interfaces for users to reanalyze public data sets without needing to download the data to their own computers. Among many other options, data sets can be reanalyzed by spectral library search (MSPLIT (21)), standard database search (MS-GF+ (22)), proteogenomics database search (Enosi (23)), spectral networks (24) and MODa (25) blind search for unexpected modifications, and spectral library search of DIA data (MSPLIT-DIA (15)). Finally, it should be noted that jPOST and PRIDE also plan to re-analyze raw MS data and re-annotate it in the future.

## UPDATES IN PROTEOMECENTRAL

Once a data set becomes publicly available (usually when the associated journal article is published), the receiving repository transmits a message coded in PX XML to the ProteomeCentral server. All metadata in the PX XML message are registered and made available in the publicly accessible ProteomeCentral portal (http://proteomecentral.proteomexchange.org). Since the original version 1.0, the PX XML schema has been updated to version 1.3 (accessible at http://proteomecentral.proteomexchange.org/doc/) to accommodate additional information related to laboratory heads responsible for data sets as a longer term contact point than the submitter and support the operations of the new PX members. The actual submitted data set files remain in the handling repository, but are linked from ProteomeCentral. Thus, PX implements a large scale, distributed database infrastructure. Users can query all PX public data sets using different biological and technical metadata, keywords, tags or references, irrespective of which original PX handling repository stores the data set files. It is important to highlight that password-protected data sets (undergoing the review process) are only accessible in the original repository, although there are instructions for reviewers on how to access them at ProteomeCentral.

The ProteomeCentral user interface has also been updated to incorporate improved summary and exploratory visualizations. There are now graphical widgets that allow users to visualize the relative proportions of data sets across all repositories by species and by instrument model, as well as word clouds that depict the most commonly repeated title words and data set-associated keywords. As the user clicks on these visualizations, the tabular listing of data sets instantly updates to reflect the selections.

## OVERALL DATA SUBMISSION AND DATA ACCESS STATISTICS

**Figure 2.**
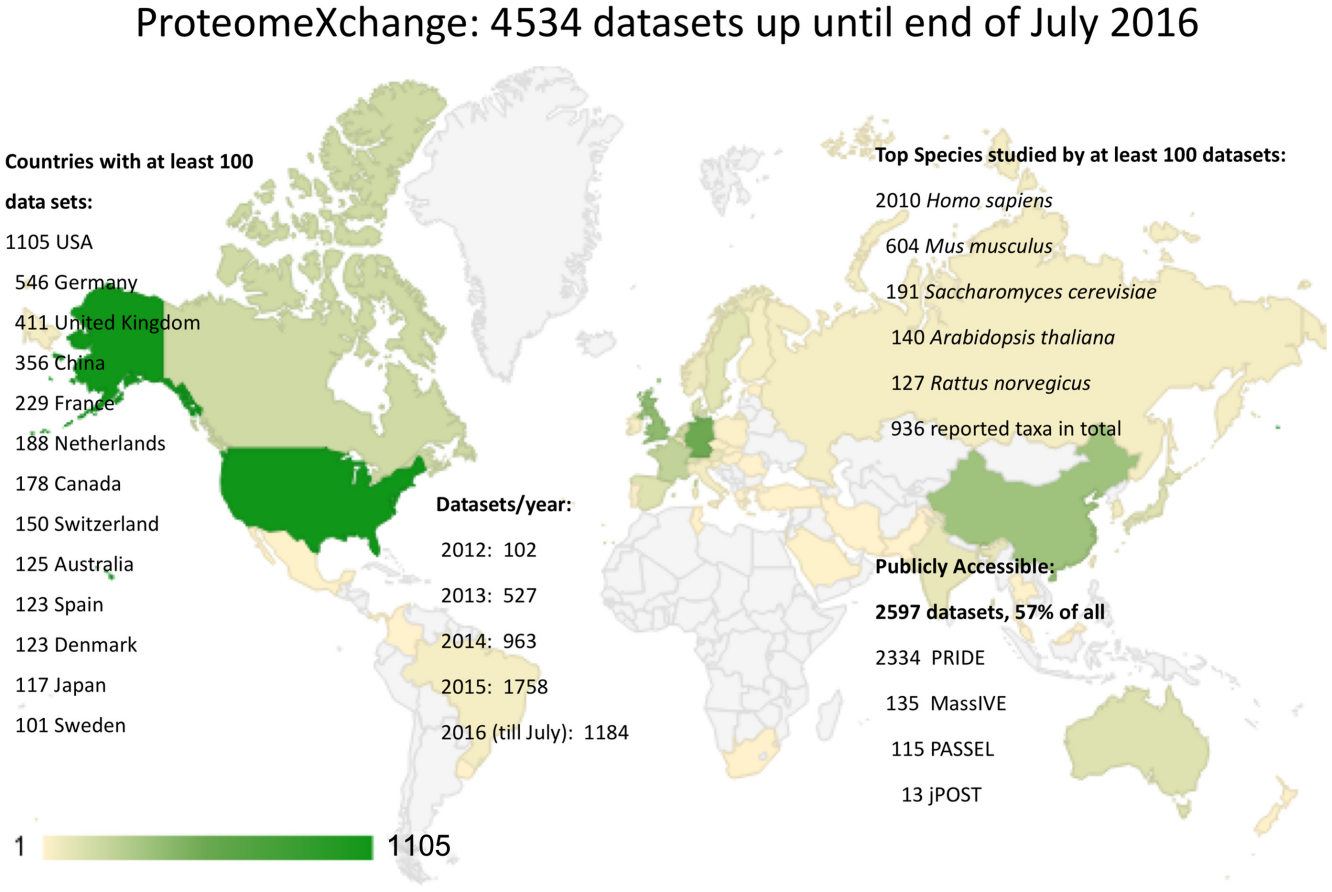
Summary of the main metrics of ProteomeXchange submitted data sets (by the end of July 2016). The number of data sets is indicated for each PX resource, data access status and for the top species and countries represented. Re-analysed data sets are not included in the metrics.

Figure 2 shows an aggregated and summary view of PX data accumulated since 2012. By the end of July 2016, a total of 4534 PX data sets had been submitted to any of the PX resources. In terms of individual resources, around 4067 data sets (representing 89.7% of all the data sets), had been submitted to PRIDE, followed by MassIVE (339 data sets), PASSEL (115 data sets) and jPOST (13 data sets, just joined PX at the beginning of July 2016). Data sets come from 50 countries, demonstrating the global reach of the consortium. The most represented countries are USA (1105 data sets), Germany (546), United Kingdom (411), China (356) and France (229). Since 2012, the number of submitted data sets has increased substantially every year, ranging from 102 (2012) to 1758 (2015). In the first seven months of 2016, a total of 1184 data sets had already been submitted.

Open data can only be open if it is made public in a timely manner. But, quite often, there is not a simple way to connect the data set to the published papers, since the authors forget to communicate that the corresponding papers to submitted data sets have been published. In the case of PX resources, 57% of these data sets (2597) were publicly available by July 2016, largely thanks to curation efforts to find papers associated to submitted data sets. It is important to highlight that the partner PX resources will make data public, without asking the original submitter, as soon as they find out that the associated paper has been published.

Overall, data sets in PX come from 936 different taxonomy IDs (Supplementary Table S1). In most cases, the information provided by the submitters is at the species level. However, terms like ‘gut microbiome’ or ‘uncultured marine microorganism‘ can be chosen as well instead of listing the actual species. The top five species (represented by more than a 100 data sets) are: *Homo sapiens* (2010 data sets), *Mus musculus* (604), *Saccharomyces cerevisiae* (191), *Arabidopsis thaliana* (140) and *Rattus norvegicus* (127). Human samples (tissues, fluids or derived cell lines) represent approximately half of all the PX data sets.

We also want to highlight that PXD accession numbers are now widely used in journal articles. PXD identifiers can be searched (using the key ‘PXD00*’) in literature resources such as PubMed, PubMedCentral and EuropePubMedCentral. As of September 2016, the numbers of publications referring to PX data sets were 558, 548 and 1426, respectively. The reason for the difference is that PubMed searches include only the text of the abstracts, while the other two resources search the full text of the articles.

## PROTEOMEXCHANGE AND THE HUMAN PROTEOME PROJECT DATA GUIDELINES

The Human Proteome Project (HPP) is an international effort by the Human Proteome Organization to expand our understanding of the entire complement of proteins encoded by the human genome. In 2012, the HPP released version 1.0 of its data guidelines (http://thehpp.org/guidelines) in which all data published in support of the HPP was required to be submitted to a PX repository. This was the first major entity specifically to make PX deposition mandatory. In a recently revised and expanded set of version 2.1 guidelines (26), PX deposition continues to be mandatory, now at the ‘complete’ submission level. This HPP deposition requirement has greatly contributed to increasing the data deposition rates of human proteome related data in the community as a whole.

## DISCUSSION AND FUTURE PLANS

In the past few years, the PX consortium has established itself as a community-responsive set of reliable proteomics data repositories to enable data submission and dissemination of MS proteomics experiments. With support from funding agencies and scientific journals, PX is actively changing the data sharing culture in the field by promoting and enabling sharing of proteomics data in the public domain. For example, after assessing the stability of PX resources, since July 2015, the journal *Molecular and Cellular Proteomics*, one of the most prominent proteomics scientific journals, is now again mandating deposition of raw data with every submitted manuscript (27), and other journals are moving in this direction (e.g. journals from the *Nature* and *PLOS* groups). In this context, public data availability and sharing in the field are quickly becoming the norm, as demonstrated by the high data submissions statistics.

Sustainability of publically accessible repositories is a crucial factor in the PX Consortium. Consider the impact of the closure of Tranche and Peptidome due to the cessation of NIH funding and the partial loss of both data sets and widespread public confidence had on the proteomics community. To ensure long-term data availability, the PX members have committed to import the data available in any other of the PX resources if it has funding problems and must cease its operations (see PX Consortium agreement at http://www.proteomexchange.org/docs/pxcollaborativeagreement.pdf), as it happened in the past regrettably for proteomics resources such as Tranche (whose thousands of surviving data sets were imported into MassIVE) and Peptidome (whose data sets were imported into PRIDE) (28), before PX was formally started. As an additional incentive toward sustainability, data will also be reproduced across repositories to enable systematic reanalysis, as MassIVE currently illustrates with tens of terabytes in hundreds of data sets already imported from PRIDE for various types of data reanalysis.

In the future, we will work on supporting ‘complete’ submissions for other popular tools and increasingly popular data workflows in the field, such as DIA (MassIVE is already starting to support some DIA data workflows at present), top down and MS imaging approaches. MaxQuant and ProteomeDiscoverer^®^ are two widely used tools for DDA shotgun proteomics workflows that cannot be supported as ‘complete’ submissions. This consortium will continue to liaise with the developers of these tools to solve interface issues and arrive at a seamless submission process. In terms of supporting other data workflows, this will involve in parallel the development of suitable open data standards.

It is also expected that reanalysis of existing data sets will only increase. In addition to PeptideAtlas, MassIVE has already started to integrate originally submitted and re-analyzed results. Better support for submitted quantification results is also on the list of priorities. At present, the files containing quantitative information are made available to download, but cannot be properly integrated in the resources. One possible solution is to achieve mzTab support by the most popular proteomics analysis tools.

Integration of proteomics data set with other ‘omics data sets is a key area where easy public accessibility of data sets will accelerate progress in the field. In this context, the PX partners are actively contributing to the recently developed OmicsDI (Omics Discovery Index) portal (http://www.omicsdi.org). The objective of OmicsDI is to provide a centralized access point to omics data sets coming from different omics approaches including genomics, transcriptomics, proteomics and metabolomics. For instance, among many other features, in OmicsDI it is possible to find different types of omics data sets associated to the same publication.

In parallel, interaction between proteomics and metabolomics resources is expected to increase, since MS is the main technology used in both fields. In fact, PSI data standards are being extended and are increasingly used to support metabolomics information (e.g. mzML and mzTab). As a concrete example, GNPS (29) is a natural products and metabolomics knowledge base implemented using the storage and computational infrastructure of the MassIVE repository. In addition to enabling the dissemination of terabytes of data in hundreds of non-proteomics MS data sets, GNPS further enables online data reanalysis and sharing of identification results toward a reusable community-wide knowledge base. The MassIVE foundation of GNPS and GNPS's demonstration of community-enabled big-data research illustrate the synergistic interactions that have already resulted from inter-omics developments in the realm of MS data sharing repositories.

It is important to note that the consortium remains open to accept new members. In this context, one resource that may join in the near future is iPROX (http://www.iprox.cn/), supported by the Beijing Proteome Research Center. Finally, we want to highlight that up-to date documentation is linked from the PX website (http://www.proteomexchange.org/). For regular announcements of all the new publicly available data sets, users can follow our Twitter account (@proteomexchange) or subscribe to the following RSS feed (https://groups.google.com/forum/feed/proteomexchange/msgs/rss_v2_0.xml).
